# Biological and Genomic Insights into *Fusarium acuminatum* Causing Needle Blight in *Pinus tabuliformis*

**DOI:** 10.3390/jof11090636

**Published:** 2025-08-29

**Authors:** Linin Song, Yuying Xu, Tianjin Liu, He Wang, Xinyue Wang, Changxiao Fu, Xiaoling Xie, Yakubu Saddeeq Abubakar, Abah Felix, Ruixian Yang, Xinhong Jing, Guodong Lu, Jiandong Bao, Wenyu Ye

**Affiliations:** 1 China National Engineering Research Center of Juncao Technology, College of JuncaoScience and Ecology, Fujian Agriculture and Forestry University, Fuzhou 350002, China; llsong2332@outlook.com (L.S.);; 2 Fujian University Key Laboratory for Plant-Microbe Interaction, College of Life Sciences, Fujian Agriculture and Forestry University, Fuzhou 350002, China; xu0yuying@foxmail.com (Y.X.);; 3 College of Plant Protection, Fujian Agriculture and Forestry University, Fuzhou 350002, China; 4 Department of Biochemistry, Faculty of Life Sciences, Ahmadu Bello University, Zaria 810281, Nigeria; 5 School of Life Sciences and Health Engineering, Luoyang Institute of Science and Technology, Luoyang 471002, China; 6 Xi’an Greening Management Center, Xi’an 710007, China; 7 Key Laboratory of Agricultural Microbiome, The Ministry of Agriculture and Rural Affairs (MARA), The Institute of Plant Protection and Microbiology, Zhejiang Academy of Agricultural Sciences, Hangzhou 310021, China

**Keywords:** Chinese pine, fungal isolation, pathogen identification, phytopathogenic fungi, biological characteristics, genome

## Abstract

Chinese pine, *Pinus tabuliformis*, is one of the most important garden plants in northern China, and the planting of this species is of great significance for the improvement of the ecological environment. In this study, different fungi were isolated and purified from diseased *Pinus tabuliformis* samples collected in Xi’an city, Shaanxi Province. Of these fungal isolates, only one (isolate AP-3) was pathogenic to the healthy host plant. The pathogenic isolate was identified as *Fusarium acuminatum* by morphological characteristics and *ITS* and *TEF-1α* sequence analyses. The optimal growth conditions for this isolate were further analyzed as follows: Optimal temperature of 25 °C, pH of 11, soluble starch and sodium nitrate as the most preferred carbon and nitrogen sources, respectively. By combining Oxford Nanopore Technologies (ONT) long-read sequencing with Illumina short-read sequencing technologies, we obtained a 41.50 Mb genome assembly for AP-3, with 47.97% GC content and 3.04% repeats. This consisted of 14 contigs with an N50 of 4.64 Mb and a maximum length of 6.45 Mb. The BUSCO completeness of the genome assembly was 98.94% at the fungal level and 97.83% at the Ascomycota level. The genome assembly contained 13,408 protein-coding genes, including 421 carbohydrate-active enzymes (CAZys), 120 cytochrome P450 enzymes (CYPs), 3185 pathogen-host interaction (PHI) genes, and 694 candidate secreted proteins. To our knowledge, this is the first report of *F. acuminatum* causing needle blight of *P. tabuliformis*. This study not only uncovered the pathogen responsible for needle blight of *P. tabuliformis*, but also provided a systematic analysis of its biological characteristics. These findings provide an important theoretical basis for disease control in *P. tabuliformis* and pave the way for further research into the fungal pathogenicity mechanisms and management strategies.

## 1. Introduction

*Pinus tabuliformis*, also known as the Chinese pine, belongs to the subgenus *Pinus* of the family Pinaceae. It is found in northern and central China, including the provinces of Shaanxi, Sichuan, Chongqing, Hubei, and Hunan [1,2,3,4]. Forest ecosystems, especially in boreal and temperate regions, are mainly dominated by conifers and form the backbone of forest ecosystems [5]. The Chinese pine is one of the most important coniferous trees in northern China and its cultivation is of great significance for improving the ecological environment [6]. It is a distinctive and evergreen coniferous species and is ideal for afforestation in China due to its tolerance to low temperatures, drought, poor soil and harsh environments [2]. This forest plant is valuable both economically, in terms of its oil, timber, and lumber supply, and ecologically, in terms of its water and soil conservation [5]. In addition, *P. tabuliformis* is often used in landscaping due to its ecological and high ornamental values in China. The morphology, genetic diversity, and biological characteristics of *P. tabuliformis* have been previously studied [7]. However, only a few diseases affecting *P. tabuliformis* have been reported. In recent years, the needle blight disease of *P. tabuliformis* emerged in the Xi’an city of Shaanxi Province, and has become severe, leading to the death of many pine trees.

Pine needle blight disease affects both the economic and ornamental value of pine trees by tissue necrosis of the needles resulting in color change [8]. This disease is one of China’s major ecological problems and has damaged millions of pine trees [9,10]. It is a serious fungal disease that affects both natural and planted pine forests in China, negatively impacting the growth and productivity of the trees [9,10]. It can cause yellowing of needles, premature needle shedding, and a decline in growth rate [9,10]. The disease first appears on the upper part of the needles. As it progresses, the needles change color [9,10].

Many pathogens have been reported to cause damage to *Pinus* spp. in worldwide. Pine forests, for example, were threatened by outbreaks of pine twig blight caused by the fungus *Pestalotiopsis trachicarpicola* [11]. The needle blight caused by *Fusarium proliferatum* has been detected in *Pinus thunbergii* [12]. The fungi *Lecanosticta acicola* and *Aureobasidium pullulans* can cause brown spot disease of *Pinus thunbergii* [13]. *Dothistroma septosporum* attacks *Pinus* spp., causing Dothistroma needle blight [14]. In addition, *Neocosmospora silvicola* was confirmed to be pathogenic to *Pinus armandii* [15]. As *P. tabuliformis* is an important species of tree for the economy, previous research has focused on breeding it, but information on the various pathogens that affect this species of pine is limited.

As a plant pathogen, *Fusarium*, a cosmopolitan genus of filamentous ascomycete, is widely distributed worldwide [16]. Some species of *Fusarium* are known to infect both plants and animals [17]. For plants, *Fusarium* is one of the most important genera of plant fungal pathogens in the world. Most *Fusarium* species can produce diverse mycotoxins that have different toxic effects on plants [18].

*Fusarium acuminatum* is a fungus that is distributed around the world and has a wide range of hosts, including animals and plants [19]. It belongs to the *Fusarium tricinctum* species complex, alongside *Fusarium avenaceum*, *Fusarium tolurosum*, and other species [19]. It causes diseases in vegetables, fruit, herbs and ornamental trees. It is also the causal agent of root rot, leaf spot, leaf blight, fruit rot, bulb rot and moldy cores in many host plants. For instance, *F. acuminatum* can cause root rot and leaf blight of *Dianthus chinensis* [20]. It also causes bulb rot of *Allium sativum* (garlic) [21], leaf blight of garlic [22], grass pea [23], and onion [24], Fusarium head blight (FHB) of wheat [25], leaf spot of *Saposhnikovia divaricata* [26], root rot of *Scutellaria baicalensis* [27], onion basal rot [28], and muskmelon leaf spot [29]. Moreover, there are few reports of *F. acuminatum* causing disease in pine trees. *F. acuminatum* can cause damping-off in *Aleppo pine* [30]. However, to the best of our knowledge, *F. acuminatum* has never been reported to cause disease in *P. tabuliformis*. The aim of this study was to identify one of the pathogens responsible for causing needle blight disease in *P. tabuliformis* in this region. Following standard analyses (including morphological features and molecular analysis), *F. acuminatum* was detected as a causal agent of this devastating disease of global concern.

## 2. Materials and Methods

### 2.1. Sample Collection and Fungal Isolation

Field observations were conducted from 2017 to 2019, in the main urban street greenbelt of Xi’an City, Shaanxi province, China. A random five-point sampling method was used, with a distance of 40 m between points and 10 samples taken from each point. Symptomatic needles were typically randomly selected for pathogen isolation. Samples of plant tissue were collected from infected pine needles. These samples were collected from 50 infected trees in five urban street green belts and transported to the laboratory.

Several representative pine needles with obvious symptoms such as drying were selected. The conventional plant tissue isolation method was used to isolate the pathogenic fungi [31]. Briefly, infected needles were surface-sterilized with 75% ethanol for 30 s, rinsed in sterile water by autoclaving and dried on blotting paper. The samples were cut into pieces of about 0.5 cm in length from the intermediate area of the diseased and healthy portions. Subsequently, the diseased pine tissues were inoculated on 2% potato dextrose agar (PDA) medium plates for fungal growth. The plates were incubated at 28 °C in the dark for 3 to 7 days. Mycelia were then sub-cultured on new PDA (potato dextrose agar) plates for further isolation and purification. Pure cultures were obtained through the isolation of single colonies on new PDA plates. Mycelial colonies were grouped according to their morphology and colony growth ratio, and microscopic characteristics. The isolates were stored on PDA slants at 4 °C for further study.

### 2.2. Pathogenicity Tests on Host Needles

A total of 50 pine needle samples were collected and 30 isolates exhibiting fungal characteristics were preliminary phenotype analysis and designated AP-1 to AP-30. All pathogenicity tests were carried out based on a method described by Xu et al. [32]. The pathogenicity of 30 isolates was tested in vitro on wounded needles of healthy Chinese pine. Mycelial discs (5 mm in diameter), from the edges of 10-day-old isolated fungal colonies, were placed on the surface of the detached needle samples, ensuring the mycelia directly contacted the plant tissues. Needles inoculated with PDA without fungal mycelium were used as controls, respectively. To maintain high relative humidity, all inoculated leaves were placed in the culture dish with wet filter paper and then incubated at 28 °C for 14 days. To further test the pathogenicity of strain AP-3, we performed an inoculation experiment using a spore suspension. We punched the spore suspension from the isolate and inoculated it onto healthy pine needles. After 28 days, we observed the incidence of needle blight disease. Briefly, three-year-old healthy *P. tabuliformis* seedlings were selected for the pathogenicity test of the AP-3 isolate. The AP-3 isolate was first cultured in liquid CMC for 3 days at 28 °C with constant shaking at 150 rpm to produce conidia. The liquid culture was then filtered to collect the spores. The spores were further suspended in sterilized distilled water and their concentration adjusted to 5 × 10^4^ conidia/mL. A total of 50 healthy *P. tabuliformis* needles per isolate were mildly injured using sterile needles. Then, the wounded sites were inoculated with equal volume of the conidia suspension (5 × 10^4^ conidia/mL) and the control plant was treated with sterile water. The inoculated and the control seedlings were covered with plastic wraps to keep them humid, then placed in a greenhouse at 25 ± 2 °C with 90% relative humidity (RH). They were observed periodically for 28 days.

### 2.3. Molecular Identification and Phylogenetic Analysis of Fungal Isolate

An isolate was selected for further molecular identification using the internal transcribed spacer (*ITS*) and Elongation factor 1-alpha (*TEF-1α*) methods [33]. Briefly, genomic DNA was extracted from the fungal mycelia on PDA plates using the CTAB method [34]. Pairs of primer specific for the *ITS* and *TEF-1α* were used to amplify the gene sequences, respectively. The amplified *ITS* and *TEF-1α* genes were run on 1% agarose gel, purified, sequenced, and analyzed for species identity. The sequencing was done at the Tsingke Biotech, Fuzhou, China, and analyzed by BLAST 2.17.0 (Basic Local Alignment Search Tool) search at the NCBI (National Center for Biotechnology Information) database (https://www.ncbi.nlm.nih.gov/), accessed on the 16 April 2025. The sequences were deposited in the NCBI GenBank database with the accession numbers PV875942 and PV855967, respectively. Phylogenetic analysis was performed based on the *ITS* and *TEF* sequences using the neighbor-joining (NJ) method. The MEGA11.0 software was used for the construction of the phylogenetic tree, using the bootstrap method with 1000 replicates [35]. All amplified loci, primers, and PCR conditions are listed in Table 1, and the strains used in this study and their corresponding GenBank accession numbers are listed in Table 2.

### 2.4. Morphological Study

The isolate AP-3 was grown on PDA at 28 °C in the dark for seven days. Its colony morphology, conidia, and hyphal shapes of the isolate were then observed under a microscope (Olympus Corporation Co., Ltd., Tokyo, Japan).

### 2.5. Culture Characteristics of the Representative Strain

#### 2.5.1. Analysis of Growth Characteristics at Different pH Values

The optimum pH for the representative strain was assessed. The growth medium pH was adjusted using 1.0 mol/L HCl or NaOH (Sinopharm Chemical Reagent Co., Ltd., Shanghai, China). The AP-3 representative strain was inoculated on PDA medium at pH values of 5.0, 6.0, 7.0, 8.0, 9.0, 10.0, 11.0, and 12.0 for the growth assay, after which it was incubated in the dark at 28 °C. After 5 days, the colony diameter was measured using Vernier calipers (Dongguan Sanliang Measuring Tools Co., Ltd., Dongguan, China), and the colony morphologies were observed and photographed. All experiments were repeated four times.

#### 2.5.2. Evaluation of Optimum Temperature

We evaluated the optimal temperature for the AP-3 isolate further by inoculating it on PDA plates and incubating the plates in the dark at temperatures of 15 °C, 20 °C, 25 °C, 30 °C, and 35 °C for 7 days. Colony growth was determined by measuring and comparing the colony diameters on the medium with Vernier calipers. All experiments were repeated four times.

#### 2.5.3. Analysis of Preferred Carbon and Nitrogen Sources

CA (Czapek) medium was used as the basic medium [50]. The carbon sources evaluated were α-Lactose (21.23 gL^−1^), maltose (30 gL^−1^), glucose (31.58 gL^−1^), soluble starch (31.06 gL^−1^), and sucrose (30 gL^−1^). The nitrogen sources evaluated were sodium nitrate (3 gL^−1^), ammonium nitrate (1.41 gL^−1^), ammonium sulfate (2.33 gL^−1^), urea (1.06 gL^−1^), and peptone (3.41 gL^−1^). Mycelium plugs (approximately 5 mm in diameter) from the AP-3 isolate were inoculated into the center of the CA medium plates containing the different carbon or nitrogen sources. sources. The plates were then cultured at 28 °C for 5 days. All experiments were repeated four times. Colony diameters were then evaluated and compared using Vernier calipers. All experiments were repeated four times.

### 2.6. Whole-Genome Sequencing and Assembly

#### 2.6.1. DNA Extraction

The fungal mycelium was harvested from a 4-day-old potato dextrose broth (PDB) culture for DNA extraction using the MolPure^®^ Fungal DNA Kit (18812ES50; Yeasen Biotechnology (Shanghai) Co., Ltd., Shanghai, China) according to the manufacturer’s protocol. The quantity and purity of the genomic DNA were analyzed using a Nanodrop (Eppendorf BioPhotometer^®^ D30; Eppendorf AG). High-quality DNA (OD260/280 = 1.8~2.0, >15 µg) was used for whole-genome sequencing.

#### 2.6.2. Genome Sequencing and Assembly

The extracted genomic DNA was sequenced using a combination of Oxford Nanopore Technologies (ONT) PromethION sequencing platform and the Illumina HiSeq 4000 sequencing platform (Biomarker Technologies Co., Ltd., Beijing, China). Genome size was estimated using GenomeScope v2.0 [51].

A de novo draft genome assembly was firstly generated by NextDenovo v2.4.0 (https://github.com/Nextomics/NextDenovo, accessed on 15 October 2021, expected genome size 42 Mb, seed reads ≥ 22,408 bp, seed depth 45×) using ONT long reads. Then, the base error correction of the draft genome assembly was conducted by NextPolish v1.3.052 [52], using both ONT and Illumina genome sequencing reads. Genome completeness was evaluated using BUSCO v5.1.2 [53] and mapping rate of short genomic sequencing reads using BWA v0.7.17-r1188 [54]. A de novo identification of repeats was conducted using RepeatModeler v2.02 (http://www.repeatmasker.org/RepeatModeler/, accessed on 20 October 2021), which generated a repeat library used for repeat masking by RepeatMasker v4.1.2 (http://www.repeatmasker.org/, accessed on 20 October 2021).

#### 2.6.3. Gene Prediction and Functional Annotation

The data generated from PacBio and Illumina platforms were used for bioinformatics analyses. The repeat-masked genome assembly was used for identification of protein-coding genes (PCGs) handled by BRAKER v2.1.6 [55], which integrated evidences from RNA-seq data and fungal homologous proteins (fungi_odb10, https://busco-data.ezlab.org/v5/data/lineages/, accessed on 08 January 2024) using Augustus v3.4.0 [56] and GeneMark-ET [57]. PCGs were functionally annotated either by InterProScan v5.52-86.0 [58] with Pfam database (9765, 72.83%), or by eggNOG-mapper v2 [59] with databases including Gene Ontology (GO; 4321, 33.12%), Kyoto Encyclopedia of Genes and Genomes (KEGG; 4788, 36.70%), and EuKaryotic Orthologous Groups (KOG; 10,166, 77.91%). A set of pathogenicity-related genes were analyzed: carbohydrate-active enzymes (CAZys) annotated by dbCAN2 [60], cytochrome P450 enzymes (CYPs) from Pfam annotation, pathogen-host interaction genes (PHI-basev4.12, http://www.phi-base.org/, accessed on 31 July 2025), and candidate secreted proteins by Pacific BioSciences (PacBio). A comparative genomic analysis was conducted using OrthoVenn3 (https://orthovenn3.bioinfotoolkits.net/, accessed on 31 July 2025) to examine the orthologous clusters and genome collinearity, between the *F. acuminatum* strain AP-3 and four other closely related species *F. graminearum*, *F. oxysporum*, *F. solani*, and *F. acuminatum*.

#### 2.6.4. Statistical Analysis

Statistical analysis and graphing were conducted using GraphPad Prism version 9.0 (GraphPad Software, San Diego, CA, USA). One-way analysis of variance (ANOVA) and the least significant difference (LSD) were utilized to evaluate the levels of significance among the samples. All experiments were conducted in triplicate.

## 3. Results

### 3.1. Natural Symptoms and Pathogen Isolation

**Figure 1:**
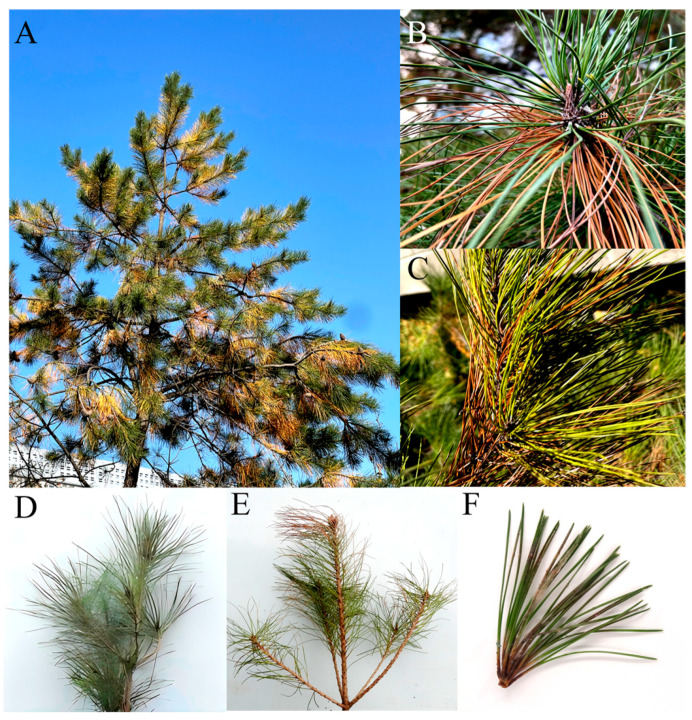
Natural symptoms under field condition and typical symptoms of inoculated diseased pine trees observed in a greenhouse: (**A**) Pine needle blight of whole tree in field; (**B**,**C**) magnified image showing symptoms on needles; (**D**) control; (**E**,**F**) pine needle blight disease symptoms caused by AP-3 isolate.

Many Chinese pine trees showed the symptoms of Chinese pine needle blight from 2017 to 2019 in Xi’an City, Shaanxi Province, China (Figure 1A–C). It was found that one to two out of every 10 trees are affected by the disease. The high temperature (average temperature: 26.4 °C) and high relative humidity (71%) environment in July create favorable conditions for disease to occur. In the early stages of infection, the plant needles turned yellow from the top to the bottom of the needles and became dry at the bottom. In the later stages of the disease, the pine needles become severely damaged and turn dark brown (Figure 1A–C).

### 3.2. AP-3 Isolate Is Pathogenic on P.tabuliformis

One strain AP-3 exhibiting the symptoms were obtained from 30 isolates. To further test the pathogenicity of strain AP-3, we punched the spore suspension from the isolate and inoculated it onto healthy pine needles. After 28 days, we observed the incidence of needle blight disease (30 inoculated needles) (Figure 1D–F). The results showed that the AP-3 isolate was the only one that was pathogenic to *P. tabulaeformis*, which is consistent with the observed field symptoms and the presence of yellow-brown lesions (Figure 1E,F). Needles inoculated with sterile water did not exhibit symptoms of needle blight (Figure 1D).

To confirm Koch’s postulates, the pathogens were successfully re-isolated from the infected needles, and their morphological characteristics and gene sequences matched those of the original isolates.

### 3.3. Identification of the AP-3 Isolate

**Figure 2:**
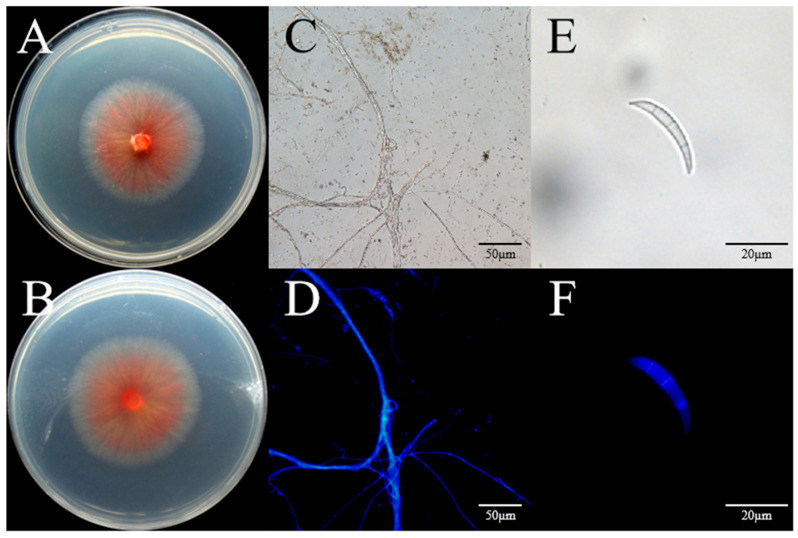
Morphological features of *Fusarium acuminatum*. (**A**,**B**) Colony morphologies (front and back) of selected isolate (AP-3) on PDA medium incubated at 25 °C for 5 days. (**C**) Mycelium of isolate without CFW (calcofluor white) treatment on PDA after 72 h incubation at 28 °C. (**D**) CFW-treated mycelium of isolate on PDA medium after 72 h incubation at 28 °C. Mycelial morphology was imaged under an Olympus-BX53F (Olympus Corporation Co., Ltd., Tokyo, Japan). Scale bar 50 μm. (**E**) Macrospore (without CFW treatment) of representative strain AP-3 after 3 days of incubation on CMC medium at 28 °C. (**F**) Macrospore with CFW treatment. Spores are falcate, slightly curved, tapering toward both ends, and have 3 septa. Scale bar = 20 µm.

The AP-3 isolate was characterized by slow-growing, pale pink colonies with a growth rate of 5 mm/day on PDA medium (Figure 2A,B). The fungal colony was round with smooth edges. At the initial stage, the fungal mycelia were white, which slowly turned into carmine from the center after 5 days of growth on PDA medium (Figure 2C,D). Microscopic observation revealed that the macroconidia were sickle-shaped with three to five septa (Figure 2E,F). Based on the morphological characteristics of AP-3, the fungus was preliminarily identified as a *Fusarium* sp.

**Figure 3:**
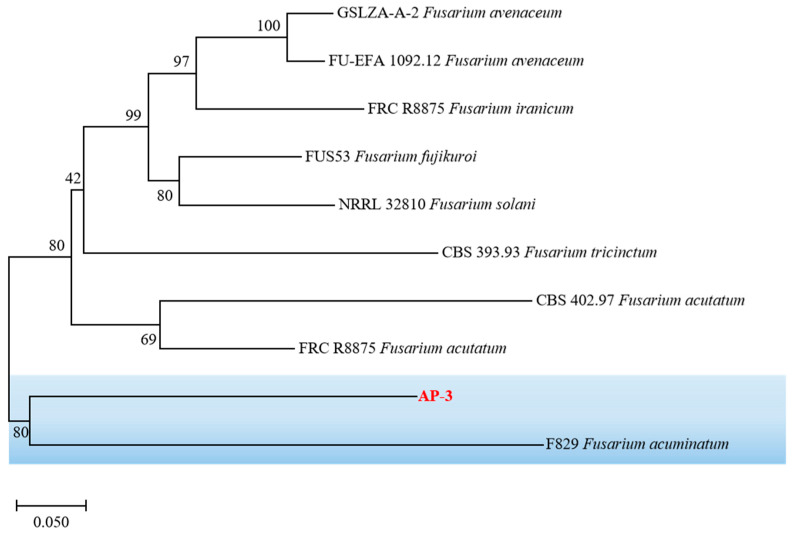
Phylogeny of selected isolates AP-3 constructed from two alignments of *ITS* and *TEF-1α* sequences. Tree is rooted in *F. acuminatum* (F829). Tree was generated by neighbor-joining method with 1000 bootstrap replicates and rooted with *F. acuminatum*. Branch values (100) are indicated and scale bar represents approximately 5% nucleotide change between close relatives.

As the AP-3 isolate was the only one pathogenic to the host plant, genomic DNA was extracted from it. The universal fungal primers ITS1/ITS4 and TEF-1/TEF2 were then used to amplify the fungal *ITS* region and the elongation factor 1-alpha region, respectively. The sequencing results were subjected to BLAST analysis in the NCBI database, after which a phylogenetic tree was eventually constructed. The AP-3 isolate was found to be most closely related to *Fusarium acuminatum* (F829), so it was identified as *F. acuminatum* (F829) (Figure 3). The *ITS* and *TEF-1α* sequences were submitted to GenBank (https://www.ncbi.nlm.nih.gov/genbank/, accessed on 29 June 2025), with the accession numbers PV875942 and PV855967, respectively. A phylogenetic tree was constructed after combining the *ITS* and *TEF-1α* sequences, and *Plectosphaerella cucumerina* (LC633900.1) was used as the outgroup. Figure 3 showed that the selected strain and the type strain *F. acuminatum* (F829) were clustered in the same branch. Therefore, based on the morphological features (Figure 2A–F) and molecular identification, we inferred that the AP-3 isolate was *F. acuminatum*.

### 3.4. Effect of pH on the Pathogen Growth

**Figure 4:**
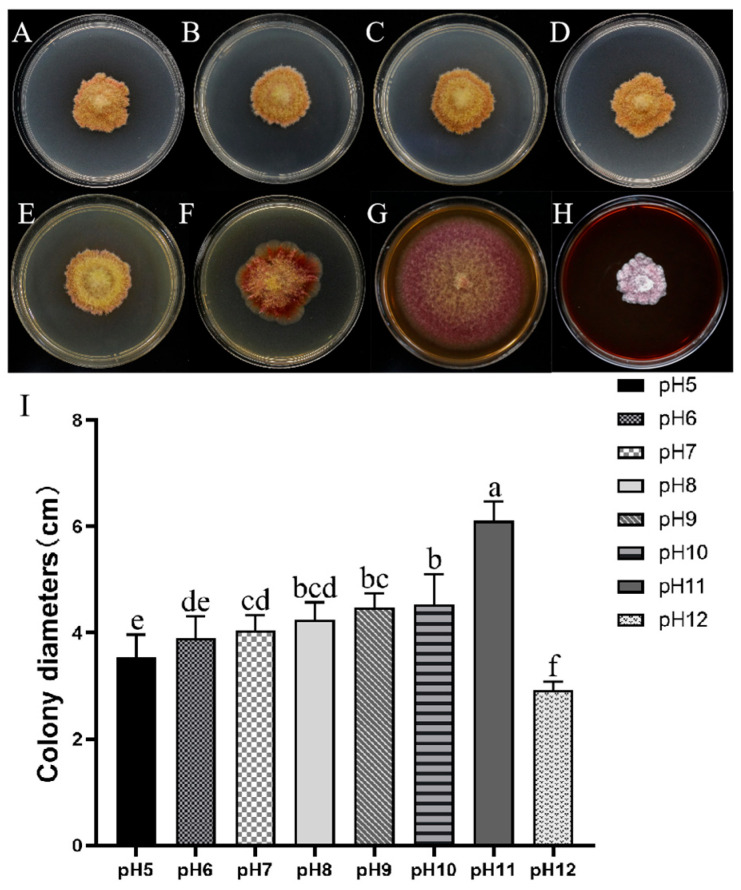
Mycelial growth of *Fusarium acuminatum* AP-3 at different pH values. (**A**–**H**) Vegetative growth of AP-3 on PDA at pH values 5.0 to 12.0 in that order. (**I**) Graphical representation of colony diameter at different pH value. Means of radial growth with standard errors were analyzed and letters indicate the significant (*p* < 0.05) differences by Duncan’s multiple range test with ANOVA using SPSS 27.0 software.

The growth of the AP-3 isolate is significantly affected by pH (Figure 4A–H). The optimum pH range for the isolate was evaluated and found to range between pH 5.0–12.0. Optimal mycelial growth was recorded at pH 11.0 with a colony diameter of 6.11 cm. This suggests that the fungus prefers an alkaline environment (Figure 4I).

### 3.5. Effect of Temperature on the Pathogen Growth

**Figure 5:**
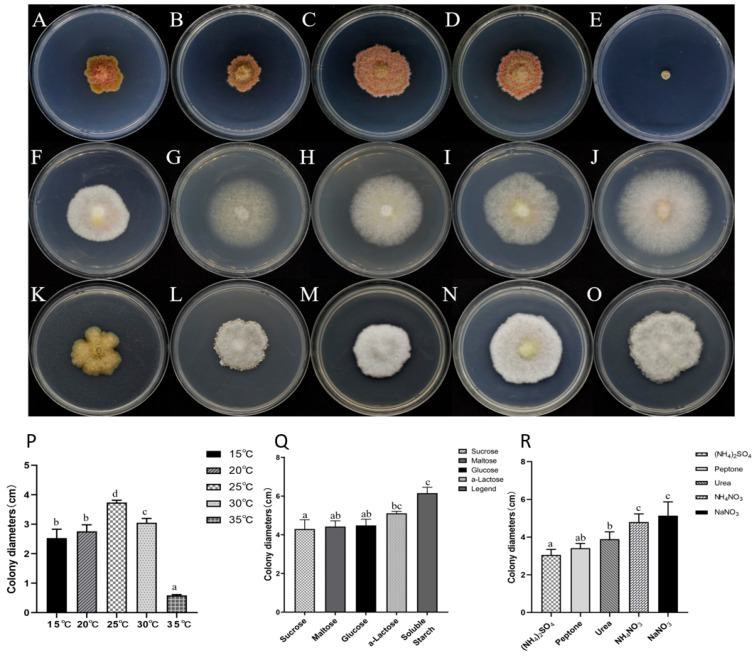
Colony morphology and diameter of representative strain AP-3 at different temperatures, carbon and nitrogen sources. (**A**–**E**) Colony morphologies of isolate AP-3 at 15–35 °C on PDA medium. (**F**–**J**) Colony morphologies of isolate. AP-3 at different carbon sources. (**K**–**O**) Colony morphologies of AP-3 isolate at different nitrogen sources. (**P**–**R**) Graphical representation of colony diameters of AP-3 isolate at different temperatures, carbon, and nitrogen sources, respectively. Values of ± standard error are represented with error bars, and different letters indicate significant differences at *p* < 0.05.

Like pH, temperature also affects the vegetative growth of the AP-3 isolate (Figure 5A–E). The optimal growth temperature was determined to be 25 °C. The strain grew at temperatures ranging from 15 °C to 35 °C, with maximum growth (colony diameter = 3.74 ± 0.07 cm) observed at 25 °C (Figure 5O). However, the AP-3 isolate hardly grows at 35 °C compared to its growth at other temperatures (Figure 5E,P).

### 3.6. Effect of Carbon Sources on the Growth of the AP-3 Isolate

The experimental results showed that the AP-3 isolate grew differently in media containing different carbon sources (Figure 5F–J). Figure 5Q shows that the fungal isolate could grow on the various tested carbon sources, but the most preferred carbon source for optimal mycelial growth was soluble starch with an average colony diameter of 6.15 ± 0.31 cm.

### 3.7. Effect of Different Nitrogen Sources on the Growth of AP-3

Figure 5K–O shows that different nitrogen sources had a significant influence on the vegetative growth of the AP-3 isolate. Although the isolate could grow on all the tested nitrogen sources (Figure 5R), sodium nitrate (NaNO_3_) was found to have the most optimal influence on the fungal mycelial growth, with an average colony diameter of 5.14 ± 0.73 cm (Figure 5R).

### 3.8. Genome Sequencing and Assembly of F. acuminatum (AP-3)

After fishing out the low-quality and short reads, the final assembled genome of the AP-3 isolate was 41.50 Mb, with 47.97% GC content and 3.04% repeats. It consisted of 14 contigs with an N50 of 4.64 Mb and a maximum length of 6.45 Mb. The genome contained 13,408 protein-coding genes, including 421 carbohydrate-active enzymes (CAZys), 120 cytochrome P450 enzymes (CYPs) from Pfam annotation, 3185 pathogen-host interaction genes (PHI), and 694 candidate secreted proteins (Table 3).

**Figure 6:**
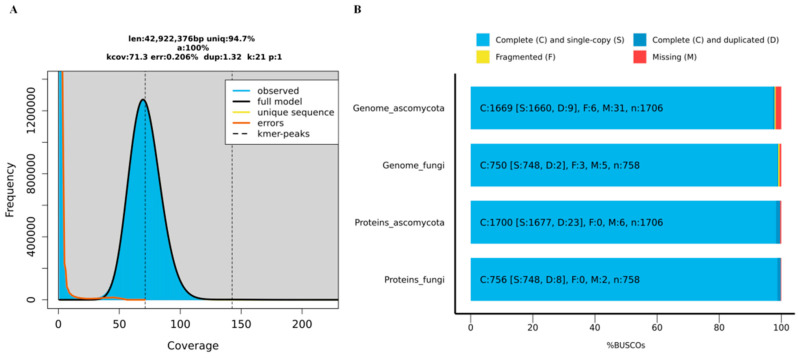
Genome features of *F. acuminatum* strain AP-3. (**A**) Estimation of genome size. Estimated by GenomeScope v2.0 with NGS short reads. (**B**) BUSCO completeness of genome assembly and predicted genes evaluated by BUSCO v5.1.2.

Genome size is a key parameter for genome assembly. Genome size was analyzed based on K-mer distribution of genomic short reads (k = 21, haploid model) [54]. The results showed that the average k-mer depth was 73 (the peak observed in Figure 6A). Also, BUSCO (Benchmarking Universal Single-Copy Orthologs) completeness analysis showed that the genome assembly for AP-3 contained 750 (98.94%) and 1669 (97.83%) complete single-copy orthologs at fungal (n = 758) and ascomycota (n = 1706) levels, respectively (Figure 6B).

We identified 13,408 PCGs, the BUSCO completeness of which was 99.74% (756/758) at fungi level, and 99.65% (1700/1706) at ascomycota level (Table 3 and Figure 6B).

**Figure 7:**
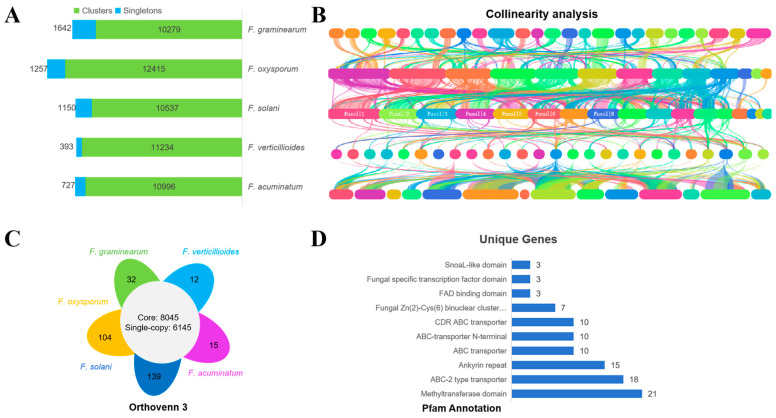
Comparative genome analysis of strain AP-3 with four closely related *Fusarium* strains using OrthoVenn3. (**A**) Orthologous clustering found 727 unique singletons in strain AP-3. (**B**) Genomic collinearity analysis using homologous clusters (Different colors represent different chromosomes). (**C**) Strain AP-3 has 15 unique clusters containing 58 genes. (**D**) Top10 Pfam items of 783 AP-3 strain-specific genes.

The *F. acuminatum* strain AP-3 was compared with four closely related *Fusarium* species (*F. graminearum*, *F. oxysporum*, *F. solani*, and *F. acuminatum*) using the OrthoVenn3 database for homologous gene analysis (Figure 7A–D). Strain AP-3 possesses 727 unique single-copy genes and 10,996 homologous gene clusters (Figure 7A). These homologous genes exhibit strong synteny across the genomes of closely related species. Notably, two contigs in AP-3 show no synteny, suggesting the presence of unique genes (Figure 7B). A total of 8045 conserved homologous gene clusters are shared between AP-3 and the four related *Fusarium* species, of which 6145 are single-copy homologous gene clusters (Figure 7C). Strain AP-3 has 15 unique homologous gene clusters (containing 58 genes) (Figure 7C), resulting in a total of 783 unique genes in AP-3. Pfam-based functional annotation revealed that these unique genes are mainly associated with the methyltransferase domain, ABC transporter domain, and fungal Zn(2)-Cys(6) binuclear cluster domain (Figure 7D).

## 4. Discussion

The Chinese pine (*P. tabuliformis*) is one of the major garden plants in northern China. Planting these trees is very important for improving the local ecology [6]. We investigated pine needle blight disease affecting *P. tabuliformis* in Xi’an City, Shaanxi Province, China. The disease had an incidence rate of 15%. Pine needle blight is emerging as a significant issue in the Chinese pine industry. In particular, it is a serious problem that needs to be solved urgently for pine growers in China. It has a significant impact on the appearance of pine trees, which seriously affects their ornamental and commodity value.

Chinese pine is an economically important garden plant that is widely cultivated in China. Due to the effects of climate change in different regions, the needle blight of pine can be caused by a variety of pathogens. For example, *Alternaria alternata* [61], *Lecanosticta acicola* [62], *Sphaeropsis sapinea* [63], *Mycosphaerella pini* [64], *Pestalotiopsis jiangsuensisi* [65], *Dothistroma septosporum* [66], *Dothistroma pini* [66], *Pestalotiopsis clavata* [67], *Pestalotiopsis chamaeropis* [67], *Pestalotiopsis lushanensis* [67], and *Pestalotiopsis neglecta* [68] have been reported to cause the needle blight disease of pine. The genus *Fusarium* has been reported in previous literature as a cause of plant diseases. For example, Fusarium dry rot disease of potatoes is caused by *F. acuminatum* [69]. However, *F. acuminatum*, as a pathogen of Chinese pine needle blight, has never been reported.

In this study, our sample collection took place in Xi’an in July. Fifty samples were not transported by cold chain, and the high temperature during transportation caused them to dry out. Despite being isolated and cultured, only 30 isolates were obtained for further experimentation. Only one (AP-3) was confirmed to be a real pathogen based on Koch’s postulates. Using wounded inoculation methods, we showed that the twenty-nine isolates were not pathogenic to needle of Chinese pine, indicating they were probably saprotrophic fungi on Chinese pine needles. The twenty-nine isolates may be pathogens, but they were not suitable for infection under greenhouse conditions. Its culture characteristics of the isolate AP-3 were studied. The morphological characteristics observed in this study were consistent with the descriptions of *F. acuminatum* by Leslie et al. [70] On PDA, the colonies grew slowly with tangerine. Macroconidia with three to five septate were formed. The isolate AP-3 produces macroconidia (Figure 2E,F), which can be transmitted by air and rain.

The *ITS* region alone cannot distinguish the species complex of *Fusarium* due to its conservation [71]. Therefore, *ITS* and *TEF1-α* gene are used to discriminate *Fusarium* species at the species level [71]. Based on morphological characteristics and molecular as well as phylogenetic analysis of two gene sequences, it was confirmed that *F. acuminatum is* the causal agent of needle blight on *P. tabuliformis* in China. To the best of our knowledge, this is the first report to implicate *F. acuminatum* as the cause of needle blight disease of Chinese pine in China. In the present study, the only one isolate (strain AP-3) of *F. acuminatum* was found in diseased *P. tabuliformis* needles. The result from our present study shows that it can not be attributed a significant role in causing *P. tabuliformis* needle disease. The reasons for a significant role in causing *P. tabuliformis* needle disease need to be studied in the future. Other factors, such as environmental factors and other pathogenic microorganisms, maybe responsible for *P. tabuliformis* needle disease in this region. Therefore, further studies are required to gain a detailed understanding of the interactions between pathogenic microorganisms and environmental factors in Chinese pine needle blight disease.

The *Fusarium* genus is widely distributed in nature, and it is one of the most important phytopathogenic fungi. Symptoms such as root rot, fruit rot, ear rot, wilt, yellowing, blight, and leaf spot caused by *Fusarium* species on various plants can lead to severe economic loss and influence ornamental value [72]. In recent years, diseases caused by *Fusarium* have gradually become a severe problem in the global cultivation of pine trees. *Fusarium oxysporum* and *Fusarium verticillioides* was confirmed as the pathogen of damping-off of pine seedlings disease in China [73]. *Fusarium circinatum* was first identified as the pathogen of pitch canker disease of some southern pines [74]. *Fusarium equiseti* and *Fusarium chlamydosporum* can cause damping-off disease to *Aleppo pine* [75]. *Fusarium chlamydosporum* was first reported as a pathogen causing damping-off disease in Algeria pine [76].

*F. acuminatum* is one of *Fusarium* species that is widely distributed around the world [77]. Favorable environment is one of the factors that promote the mycelial growth of plant-pathogenic fungi. In order to determine the conditions suitable for mycelial growth of the *F. acuminatum* strain AP-3, it is essential to investigate its characteristics such as pH value, temperature, carbon sources, nitrogen sources, and NaCl stress (Figure S1). Maintaining a constant pH was one of the most important factors for achieving optimal growth rates of *F. acuminatum*. The optimum pH for the *F. acuminatum* strain AP-3 was 11.0 (Figure 4I). However, the present study yielded some findings that differed from those of previous studies. For example, a previous study found that the most suitable pH value for *F. acuminatum* WHWNSHJ1 was 7.0 while ours showed that it was 11.0 [78]. The differences in pH value may be caused by differences in host and regions where the study was conducted. Temperature, as an important environmental factor, influences the mycelial growth of *F. acuminatum*. It has an optimal temperature of 25 °C, but limited growth occurs in 35 °C (Figure 5P). *F. acuminatum* are capable of metabolizing a wide range of carbon sources and nitrogen sources, including glucose, soluble starch, maltose, α-lactose, ammonium nitrate, ammonium sulfate, urea, and peptone. The strain AP-3 can effectively use a variety of carbon and nitrogen sources for growth, among which soluble starch and sodium nitrate were the most suitable carbon and nitrogen sources, respectively (Figure 5Q,R). The findings of previous studies on some of biological characteristics (including temperature, pH value, carbon source and nitrogen sources) of *F. acuminatum* were similar to ours. This indicates that the optimal temperature for *F. acuminatum* AP-3 mycelial growth is 25 °C [78]. The optimal carbon source is soluble starch, while the optimal nitrogen source is sodium nitrate [79]. In the case of salt stress, it reflects how well the strain can adapt to adverse conditions.

In addition, the whole genome of the *F. acuminatum* strain AP-3 was sequenced, assembled and annotated using a combination of Oxford Nanopore Technologies (ONT) long-read sequencing and Illumina short-read sequencing technologies. Our result showed that the genome size and GC content of the *F. acuminatum* strain AP-3 was 41.5 Mb and 47.97% (Table 3), respectively. To our knowledge, no information has been reported on the genome of *F. acuminatum*. The reference genome size of *F. acuminatum* 1A (GCA_038181435.1) from Medicago sativa was 46.7 Mb and the GC content was 48% in NCBI (https://www.ncbi.nlm.nih.gov/datasets/genome/GCA_038181435.1/, accessed on 15 April 2025).

Through annotation of the genome of the *F. acuminatum* strain AP-3, we found that a set of pathogenicity-related genes, including 421 carbohydrate-active enzymes (CAZys) annotated by dbCAN2 [60], 120 cytochrome P450 enzymes (CYPs) from Pfam annotation, and 3185 pathogen-host interaction genes, were identified. These genes could help us to understand its infection mechanism in future.

When a pathogen infects a host, it produces a large number of virulence factors, including effector proteins that play a crucial role in the process of pathogenesis [80]. The genome of the *F. acuminatum* strain AP-3 contains 694 genes of putative secreted proteins associated with the fungal host cell wall (Table 3). The identification of these genes provides a research basis for the subsequent development of specific fungicides against *F. acuminatum*.

Pathogenic fungi produce various secondary metabolites, including a total 51 secondary metabolite biosynthesis gene clusters (SMBGCs) were identified by fungal version of antiSMASH v5.2.0 [81] (Table 3). It included twenty-three non-ribosomal peptide synthetases (NRPSs), eight NPRS-likes, nine terpenes, seven Type I polyketide synthases (T1PKSs), three indoles, and one betalactone. In addition, there are also many unknown secondary metabolites in *F. acuminatum* AP-3, suggesting that this strain has the potential to produce bioactive compounds. It was reported that *F. acuminatum*, like other species within the *Fusarium* genus, is capable of producing various mycotoxins, including deoxynivalenol (DON), T-2 toxin and HT-2 toxin [82]. *F. acuminatum* possesses a similar mycotoxigenic profile to *F. avenaceum*, being able to biosynthesize secondary metabolites such as enniatins (ENNs) [19] and moniliformin (MON) [83]. Further studies into the pathogenicity mechanism of *F. acuminatum* and disease management should be conducted in future.

Overall, these findings can aid in analyzing the pathogenic mechanism of *F. acuminatum* infection on Chinese pine and developing effective prevention and control strategies for Chinese pine needle blights disease.

## 5. Conclusions

In summary, this study identified *F. acuminatum* as the causal agent of needle blight in pine (*P. tabuliformis*). Moreover, the pathogenicity, biological traits, and genome information of the pathogen have been established, providing critical insights for disease control. However, the pathogen’s environmental adaptability, molecular interactions with host, and potential biocontrol approaches need further investigation to develop targeted management strategies.

## Figures and Tables

**Table 1:** Amplification sites, primer sequences, and PCR conditions used in this study.

Locus ^a^	Primer	Primer Sequence	PCR Conditions	Reference
*ITS*	ITS1 ITS4	TCCGTAGGTGAACCTGCGG	94 °C for 5 min (94 °C for 40 s, 58 °C for 40 s, and 72 °C for 60 s) × 35 cycles, 72 °C for 7 min	[33]
		TCCTCCGCTTATTGATATGC		
*T* *EF-1α*	TEF1	ATGGGTAAGGAAGACAAGAC	94 °C for 3 min (94 °C for 60 s, 60 °C for 60 s, and 72 °C for 3 min) × 32 cycles, 72 °C for 5 min	[36]
	TEF2	GGAAGTACCAGTGATCATGTT		

^a^ Genes: *ITS*, internal transcribed spacer; *TEF-1α*, translation elongation factor 1-alpha.

**Table 2:** Strains used in this study.

Original Name	Culture Accession Number(s)	Type Status	Accession No. *ITS*	Accession No. *TEF-1α*	Reference
*Fusarium acutatum*	CBS 402.97	Type of *Fusarium avenaceum*	NR111142.1	MT010989.1	[37,38]
*Fusarium avenaceum*	NRRL 54939	Type of *Fusarium avenaceum*	PP336534.1	MH582391.1	[39]
*Plectosphaerella cucumerina*	SJB163	Type of *Plectosphaerella cucumerina*	LC633900.1	LC633926.1	[40]
*Fusarium avenaceum*	GSLZA-A-2	Type of *Fusarium avenaceum*	KX029335.1	KX029338.1	[41]
*Fusarium avenaceum*	FU-EFA 1092.12	Type of *Fusarium avenaceum*	PP660998.1	PP726199.1	[42]
*Fusarium acuminatum*	F829	Type of *Fusarium acuminatum*	JABEEU010000081.1(genome)		[43]
*Fusarium tricinctum*	CBS 393.93	Type of *Fusarium tricinctum*	MH862424.1	LC468080.1	[44,45]
*Fusarium fujikuroi*	FUS53	Type of *Fusarium fujikuroi*	MK630074.1	LC468051.1	[46,47]
*Fusarium iranicum*	FRC R8875	Type of *Fusarium iranicum*	OL832291.1	OL772863.1	[48]
*Fusarium solani*	NRRL 32810	Type of *Fusarium solani*	DQ094577.1	DQ247118.1	[49]

**Table 3:** Genome characteristics of *Fusarium acuminatum* strain AP-3.

Features	AP-3
ONT ^*^ long reads	5.18 Gb (~125×)
Illunima short reads	3.69 Gb (~89×)
Assembly size (bp)	41,498,617
Contig number	14
Contig N50 (bp)	4,642,861
Contig L50	4
Average contig length (bp)	2,964,186
Maximum contig length (bp)	6,454,297
GC content	47.97%
Repeat sequence	3.04%
Illumina reads mapping rate	99.10%
Protein-coding genes	13,408
Genes annotated by Pfam ^#^	9765
Genes annotated by GO ^#^	4321
Genes annotated by KEGG ^#^	4788
Genes annotated by KOG ^#^	10,166
Genes annotated by CAZy ^#^	421
Pathogen-host interaction genes	3185
Cytochrome P450 enzymes	120
Putative secreted proteins	694
SMBGCs ^$^	51

ONT ^*^: Oxford Nanopore Technologies. ^#^: Annotated by eggNOG-mapper v2 online service (http://eggnog-mapper.embl.de/, accessed on 31 July 2025). ^$^: SMBGCs (Secondary Metabolite Biosynthesis Gene Clusters) analyzed by antiSMASH v5.2.0.

## Data Availability

The genome assembly and gene annotation of *F. acuminatum* isolate AP-3 (GWHBFSS00000000) have been deposited in the GWH (Genome Warehouse, https://ngdc.cncb.ac.cn/gwh, accessed on 31 July 2025) database in National Genomics Data Center, China National Center for Bioinformation (CNCB-NGDC, https://ngdc.cncb.ac.cn/, accessed on 31 July 2025), under Bioproject PRJCA007035. The raw sequence data (CRA005282), including ONT long reads (CRR335559), NGS short reads (CRR335560), and RNA-seq reads (CRR335558), are available at the GSA (Genome Sequence Archive, https://ngdc.cncb.ac.cn/gsa, accessed on 31 July 2025).

## Supplementary Materials

The following supporting information can be downloaded at: https://www.mdpi.com/article/10.3390/jof11090636/s1, Figure S1: The strain tolerance to different salt concentrations.
