# Supplementary figures and images for: Biological and Genomic Insights into Fusarium acuminatum Causing Needle Blight in Pinus tabuliformis
Source: J Fungi (Basel). 2025 Aug 29;11(9):636. doi: 10.3390/jof11090636 (PMC12470730; doi:10.3390/jof11090636)

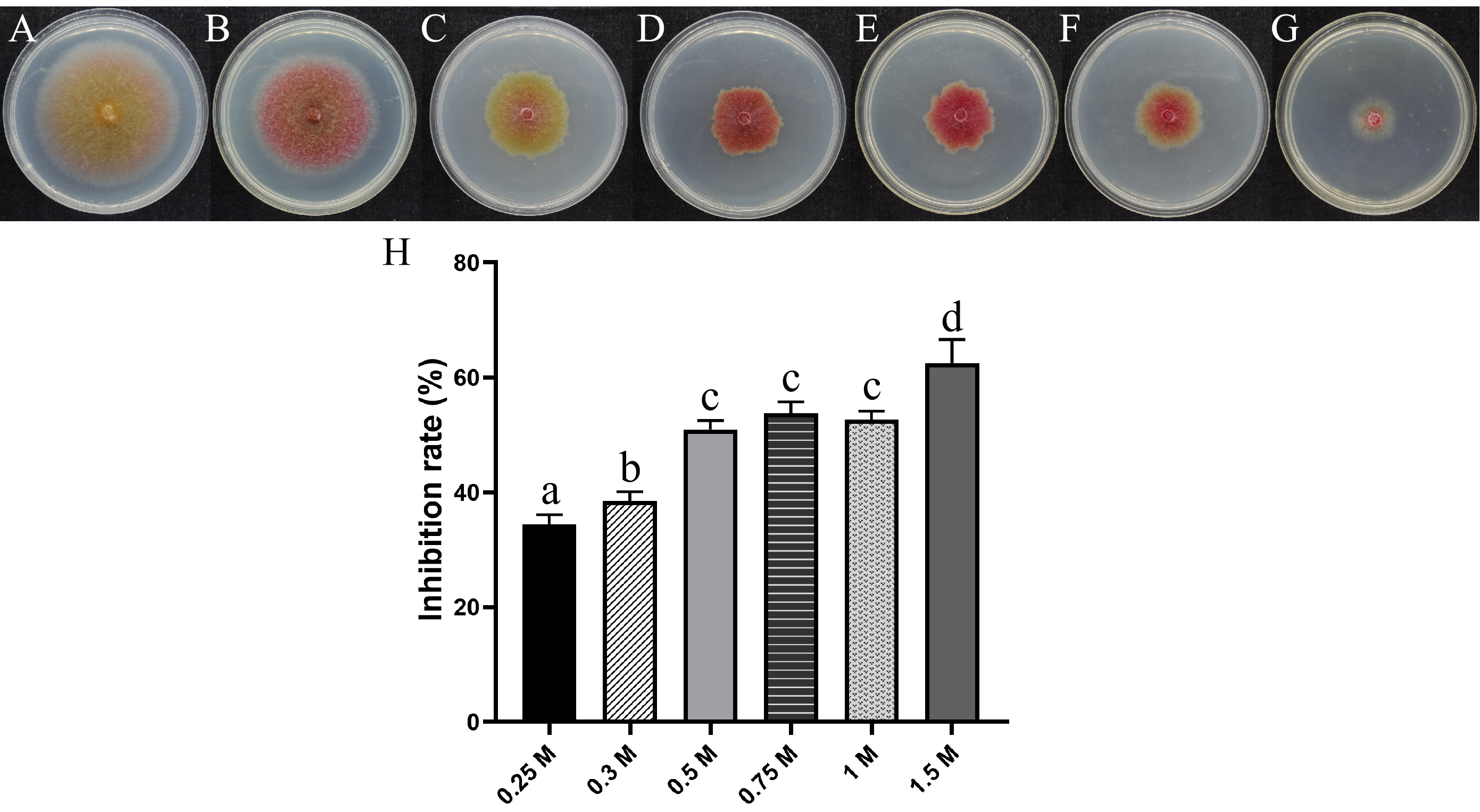

Supplement: Supplementary file 1 [file jof-11-00636-s001.zip › jof-3829657-supplementary.tif]
